# Pediatric Crohn’s Disease in Ethiopia: A Diagnostic Challenge in a High Tuberculosis Prevalence Setting

**DOI:** 10.7759/cureus.91865

**Published:** 2025-09-08

**Authors:** Binyam M Habte, Yoseph M Habte, Makida M Habte, Esimael M Abdu, Shimelis A Yimer

**Affiliations:** 1 Department of Medicine, University of Gondar, Gondar, ETH; 2 Department of Medicine, Ethio Tebib Hospital, Addis Ababa, ETH; 3 Department of Medicine, Bethel Medical College, Addis Ababa, ETH; 4 Department of Surgery, Teklehaimanot General Hospital, Addis Ababa, ETH; 5 Department of Pathology, Ethio Tebib Hospital, Addis Ababa, ETH

**Keywords:** case report, crohn’s disease (cd), ethiopia, intestinal tuberculosis, pediatric inflammatory bowel disease

## Abstract

Differentiating Crohn’s disease from intestinal tuberculosis can be particularly challenging in regions where tuberculosis is endemic and gastrointestinal symptoms overlap. We report the case of a nine-year-old Ethiopian girl who presented with a five-month history of cramping periumbilical abdominal pain and two months of intermittent bright red, blood-tinged stools, initially treated for parasitic infections and peptic ulcer disease without improvement. Physical examination was unremarkable except for mild lower abdominal tenderness. Laboratory studies, chest radiography, and abdominal ultrasound were normal. Colonoscopy revealed multiple areas of cobblestoning in the terminal ileum. Histopathology demonstrated mucosal and submucosal lymphocytic infiltrates with prominent lymphoid follicles and no granulomas. GeneXpert MTB/RIF (Cepheid, Sunnyvale, CA, USA) testing and immunohistochemistry were negative. Based on these findings, a diagnosis of Crohn’s disease was established, and the patient was started on azathioprine, prednisolone, and cotrimoxazole prophylaxis, with subsequent marked clinical improvement. This case highlights the diagnostic challenge of differentiating pediatric Crohn’s disease from intestinal tuberculosis in endemic regions and underscores the importance of maintaining a broad differential diagnosis. Timely, systematic evaluation using clinical, endoscopic, histopathological, and microbiological data can facilitate accurate diagnosis and effective management, even in resource-limited settings. Increased awareness and reporting of pediatric inflammatory bowel disease in sub-Saharan Africa are essential for earlier recognition, appropriate treatment, and improved patient outcomes.

## Introduction

Crohn’s disease (CD) is a chronic, relapsing inflammatory bowel disease (IBD) characterized by transmural inflammation that can affect any segment of the gastrointestinal tract. Although well recognized in developed countries, it appears to be uncommon in sub-Saharan Africa, especially among pediatric populations. However, this apparent rarity is likely due to significant limitations in available epidemiological data, including underdiagnosis, underreporting, and limited access to advanced diagnostic tools. Furthermore, the high prevalence of infectious enteropathies such as intestinal tuberculosis (ITB) complicates the diagnostic process, making it difficult to accurately assess the true burden of CD in these regions [1].

Distinguishing CD from ITB poses a significant clinical challenge, particularly in tuberculosis-endemic settings, as both conditions can present with abdominal pain, altered bowel habits, gastrointestinal bleeding, and endoscopic findings such as ulcerations and mucosal irregularities. Histopathological overlap, limited access to advanced diagnostic tools, and a low index of suspicion for IBD in these regions further contribute to diagnostic delays and potential mismanagement [2,3].

Here, we report the case of a nine-year-old Ethiopian girl presenting with chronic abdominal pain and hematochezia, initially suspected to have ITB. Comprehensive endoscopic and histopathological evaluation, however, confirmed a diagnosis of CD. This case underscores the importance of including IBD in the differential diagnosis of chronic gastrointestinal symptoms in pediatric patients, even in low-incidence regions, and emphasizes the need for a systematic diagnostic approach to differentiate CD from ITB in resource-limited settings.

## Case presentation

In January 2025, a nine-year-old prepubertal girl who had not yet attained menarche presented with a five-month history of cramping periumbilical abdominal pain, accompanied for the past two months by intermittent passage of bright red, blood-tinged stools. The symptoms were associated with loss of appetite but were not accompanied by diarrhea, constipation, or weight loss. She was initially evaluated at a local health center, where she received albendazole and tinidazole for a presumptive parasitic infection and later empirical treatment for peptic ulcer disease. However, there was no symptomatic improvement, and she was subsequently referred to our hospital.

On examination, her vital signs were stable, and abdominal palpation revealed bilateral lower abdominal tenderness. The remainder of the systemic examination was unremarkable. Laboratory investigations demonstrated a normal complete blood count and a negative stool ova and parasite test (Table 1). Chest radiography and abdominal ultrasonography were both normal. Given the persistence of symptoms and a clinical suspicion of intestinal tuberculosis, a colonoscopy with biopsy was performed.

**Table 1:** Laboratory results at presentation

Test	Result	Reference value
White Blood Cells	9,500/mm^3^	4,100-11,000/mm^3^
Neutrophil	72.2%	50-74%
Lymphocyte	24%	20-40%
Eosinophil	1%	0-6%
Hemoglobin	13.2 g/dl	11-16 g/dl
Platelet	263,000/mm^3^	100,000-400,000/mm^3^
Blood Urea Nitrogen	12 mg/dl	6-20 mg/dl
Creatinine	1.1 mg/dl	0.7-1.2 mg/dl
Erythrocyte sedimentation rate	16 mm/hr	0-20 mm/hr
Aspartate Transaminase	24 IU/L	Up to 31 IU/L
Alanine Transaminase	19 IU/L	Up to 45 IU/L
Hepatitis B surface antigen	Negative	
Hepatitis C antibody	Negative	
HIV rapid diagnostic test	Negative	

**Figure 1:**
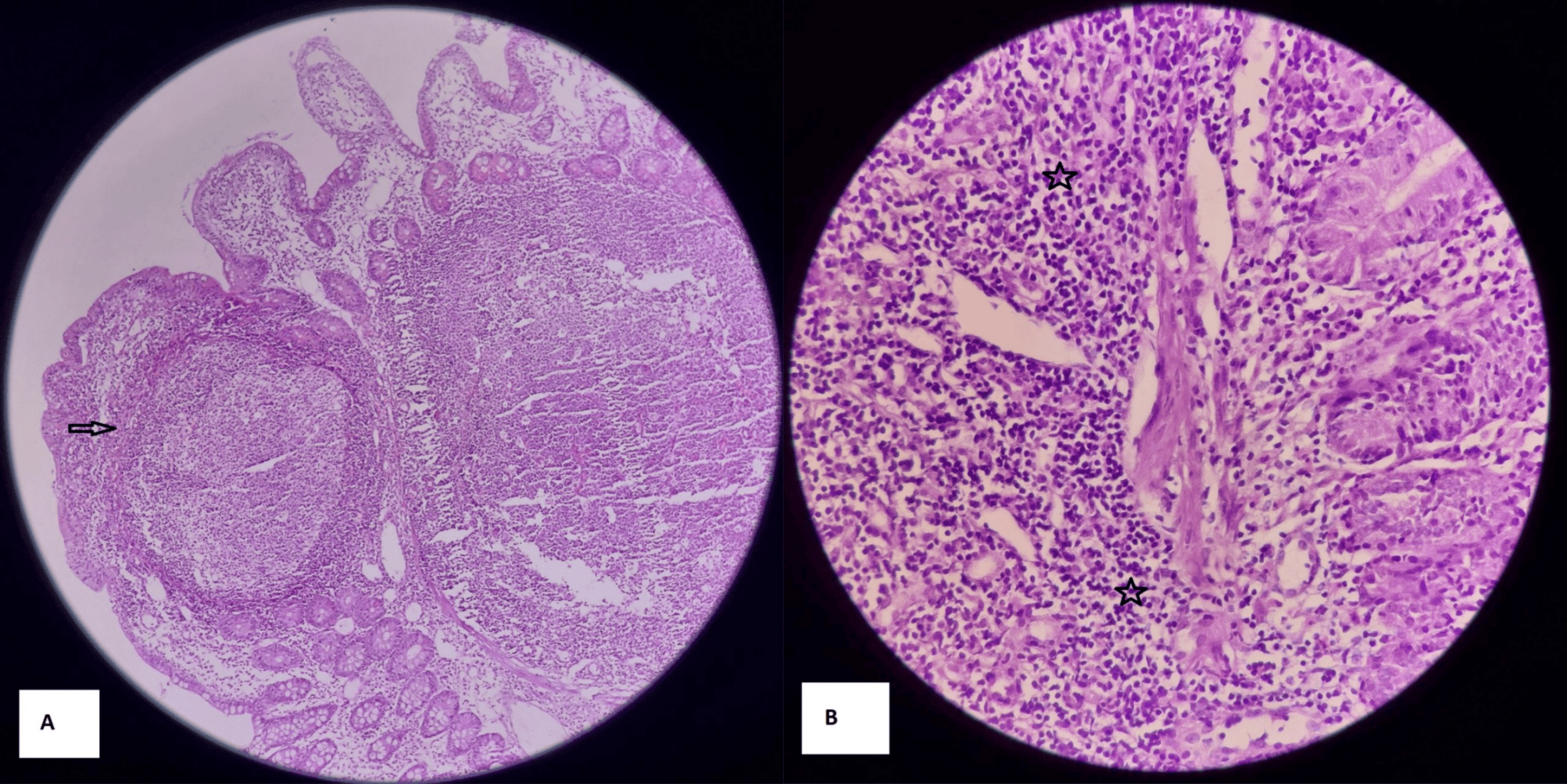
Histopathological examination of terminal ileal biopsy specimens (a) Colonic mucosa demonstrating prominent lymphoid follicle (b) Mucosal and submucosal lymphocytic infiltrates. Transmural inflammation with a mixed infiltrate of lymphocytes, plasma cells, macrophages, and neutrophils may be observed; noncaseating granulomas, when present, help distinguish Crohn’s disease from other conditions.

Endoscopic evaluation revealed multiple areas of cobblestoning in the terminal ileum. Histopathological examination of terminal ileal biopsy specimens, comprising multiple gray-white tissue fragments measuring approximately 1 cm in aggregate, demonstrated mucosal and submucosal lymphocytic infiltrates with large lymphoid follicles containing prominent germinal centers. No granulomas were identified (Figure 1). These findings were consistent with a chronic inflammatory process involving both the mucosa and submucosa, favoring a diagnosis of Crohn’s disease. GeneXpert MTB/RIF (Cepheid, Sunnyvale, CA, USA) testing for Mycobacterium tuberculosis complex was negative, and immunohistochemistry did not reveal any specific alternative pathology.

Based on the correlation of endoscopic and histopathological findings, a diagnosis of Crohn’s disease was established. The patient was initiated on azathioprine, cotrimoxazole, and prednisolone. During six months of follow-up, she showed progressive clinical improvement, with complete resolution of abdominal pain and rectal bleeding and recovery of appetite. Laboratory monitoring, including complete blood count, inflammatory markers, and liver function tests, remained within normal limits. She was regularly assessed for growth, nutritional status, and potential medication side effects. No disease flare, complications, or hospitalizations occurred during this period, and she remained compliant with her medications. The patient continues to be scheduled for regular follow-up to monitor disease activity and maintain remission.

## Discussion

CD is an uncommon diagnosis in sub-Saharan Africa, particularly among children, where infectious causes of chronic gastrointestinal symptoms predominate. This epidemiological pattern has historically contributed to the underrecognition and delayed diagnosis of IBD in the region [4,5]. In Ethiopia, the scarcity of epidemiological data further limits awareness among clinicians, and pediatric cases are rarely documented in the literature. Consequently, children with CD are often initially misdiagnosed and treated for more common local conditions such as helminthiasis, amoebiasis, or ITB, as occurred in this patient.

The clinical overlap between CD and ITB presents a substantial diagnostic challenge in tuberculosis-endemic countries. Both conditions may manifest with abdominal pain, altered bowel habits, gastrointestinal bleeding, weight loss, and systemic symptoms [2,6]. Endoscopically, features such as ulcerations, strictures, and mucosal irregularities can be shared, while histologically, both may show chronic inflammatory infiltrates, lymphoid aggregates, and architectural distortion [3,6]. Although the presence of caseating granulomas strongly favors ITB, these are not universally present, and non-caseating granulomas may also be observed in CD, further complicating the diagnostic picture [7].

In this case, the absence of granulomas, the negative GeneXpert MTB/RIF assay, and the lack of supportive microbiological evidence for tuberculosis shifted the diagnosis toward CD, consistent with previous studies emphasizing the importance of integrating clinical, endoscopic, histopathological, and microbiological findings. The characteristic cobblestoning observed on colonoscopy, together with mucosal and submucosal lymphocytic infiltrates and prominent lymphoid follicles without granulomas, further supported the diagnosis of Crohn’s disease.

This case also underscores the importance of maintaining a broad differential diagnosis for pediatric patients presenting with chronic gastrointestinal symptoms in low-incidence settings. Early consideration of IBD is essential to prevent prolonged morbidity resulting from misdiagnosis and inappropriate treatment. In regions with high TB prevalence, the use of an evidence-based diagnostic algorithm, incorporating endoscopic evaluation, targeted biopsies, molecular testing for Mycobacterium tuberculosis, and exclusion of infectious causes, remains critical [8].

From a management perspective, the patient’s marked improvement following initiation of azathioprine, prednisolone, and cotrimoxazole prophylaxis demonstrates that standard IBD therapeutic protocols can be effective in resource-limited settings, provided that the diagnosis is established early. Nevertheless, significant challenges remain, including limited availability of pediatric gastroenterology services, the high cost of biologic therapies, and the need for ongoing monitoring to detect treatment-related complications [9].

This case illustrates the considerable diagnostic challenges in distinguishing pediatric Crohn’s disease from intestinal tuberculosis in regions where TB is endemic and IBD is uncommon. It underscores the need for heightened clinical vigilance in children presenting with persistent gastrointestinal symptoms, and demonstrates that, even in resource-limited settings, timely and systematic diagnostic evaluation, including endoscopy, targeted biopsies, and microbiological testing, can enable accurate diagnosis and effective management. Broader awareness and documentation of such cases are essential to improve recognition and facilitate earlier intervention for IBD in sub-Saharan Africa.

## Conclusions

Pediatric Crohn’s disease can mimic intestinal tuberculosis, particularly in tuberculosis-endemic regions, posing significant diagnostic dilemmas. This case highlights the critical importance of maintaining a broad differential diagnosis and employing a comprehensive approach that integrates clinical, endoscopic, histopathological, and microbiological findings. Early diagnosis and initiation of appropriate therapy can achieve substantial clinical improvement, even in settings with limited resources. Enhanced reporting and dissemination of pediatric IBD cases in sub-Saharan Africa are pivotal for increasing clinician awareness, refining diagnostic strategies, and ultimately improving patient outcomes.
